# P-126. Host Factors Associated with Failure of Fecal Microbiota Transplant for Recurrent Clostridioides difficile Infection: 10- Year Real World Experience in a Dedicated C. difficile Clinic

**DOI:** 10.1093/ofid/ofae631.331

**Published:** 2025-01-29

**Authors:** Kibret Yohannes, Joseph D Nguyen, Initha Setiady, Jae Hyun Shin, Cirle A Warren, Emma C Phillips, R Ann Hays, Brian Behm

**Affiliations:** University of Virginia School of Medicine, Springfield, Virginia; University of Virginia School of Medicine, Springfield, Virginia; University of Virginia School of Medicine, Springfield, Virginia; University of Virginia, Charlottesville, Virginia; University of Virginia, Charlottesville, Virginia; Ohio State University Wexner Medical Center, Columbus, Ohio; University of Virginia, Charlottesville, Virginia; University of Virginia School of Medicine, Springfield, Virginia

## Abstract

**Background:**

*Clostridioides difficile* infection (CDI) continues to be an antibiotic-associated infection of urgent concern. Fecal microbiota transplantation (FMT) is the most effective treatment of recurrent CDI (rCDI). Despite high success rates, FMT has proven to be ineffective in 5-20% of cases. Factors associated with failure have not been clearly defined. We seek to better understand factors predictive of failure in patients who have undergone FMT.

Failure Rate by Comorbidity

**Figure:**
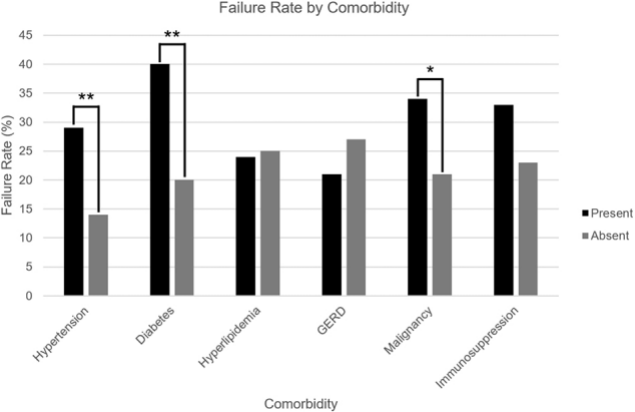

**Methods:**

A retrospective chart review was conducted on adult patients who were screened at the Complicated *C. difficile* Clinic at the University of Virginia Health System and received FMT for rCDI between 2013 and 2022. Electronic medical records were thoroughly reviewed to collect data on demographics, comorbidities, CDI history, laboratory values prior to FMT, and performance after FMT. Primary outcome was failure of FMT, defined as either rCDI or death within one year.

Failure Rate by Lab Values

**Figure:**
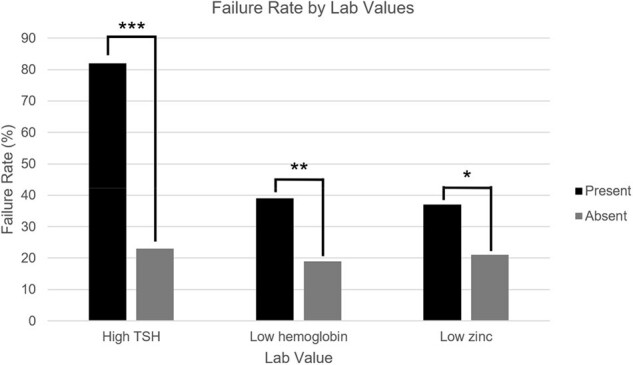

**Results:**

240 patients with rCDI were treated with FMT: 70.4% were female, median age was 68 (range: 20 to 96), and median episodes of CDI was 4 (range: 1 to 25). Within one year, 24.6% of patients experienced failure: 18.3% of patients experienced rCDI and 7.1% died. Univariate analysis showed that older age (≥70), male sex, ≥4 episodes of CDI, hypertension, diabetes mellitus, malignancy, elevated TSH, anemia, and low zinc were linked to failure. Older age, ≥4 episodes of CDI, hypertension, and elevated TSH were also associated with recurrence. Male sex, diabetes mellitus, elevated TSH, anemia, and low zinc were associated with death. Multivariate analysis showed that older age, ≥4 episodes of CDI, and diabetes mellitus were linked to failure while older age and ≥4 episodes of CDI were linked to recurrence.

Failure Rate by Number of C. difficile Infections

**Figure:**
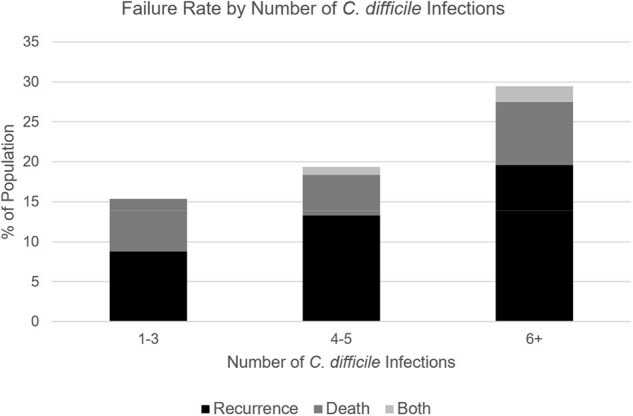

**Conclusion:**

FMT continues to hold up as an effective treatment for rCDI. At one year follow-up, about 1 in 4 patients who underwent FMT at our institution experienced failure. Older age, ≥4 episodes of CDI, elevated TSH, anemia, and low zinc appear to be associated with failure, but additional research is needed to clearly define causality. Our results underscore the importance of recognizing factors that can predict failure and be targeted to further improve outcomes of FMT.

Failure Rate by Sex

**Figure:**
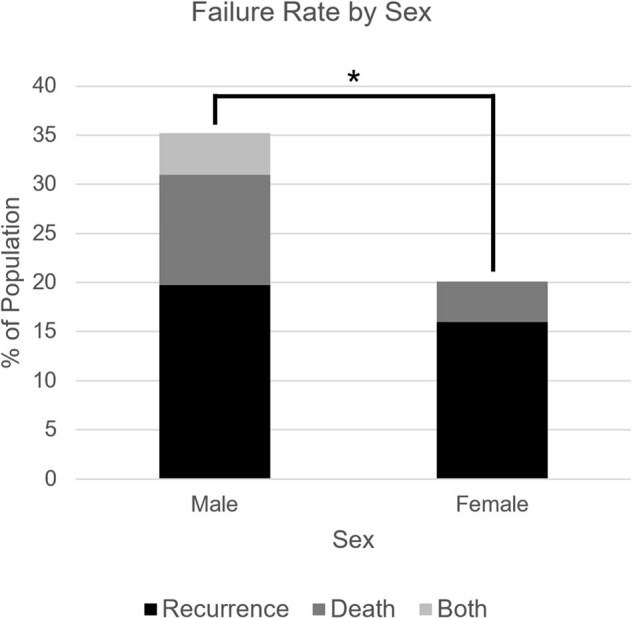

**Disclosures:**

**Jae Hyun Shin, MD**, Ferring: Grant/Research Support **Cirle A. Warren, MD**, Ferring Pharmaceuticals: Site PI for ROAR

